# Genome-wide identification, expression profiling, and functional analysis of ammonium transporter 2 (AMT2) gene family in cassava (*Manihot esculenta* crantz)

**DOI:** 10.3389/fgene.2023.1145735

**Published:** 2023-02-22

**Authors:** Jinze Xia, Yu Wang, Tingting Zhang, Chengcai Pan, Yiyin Ji, Yang Zhou, Xingyu Jiang

**Affiliations:** ^1^ National Center of Technology Innovation for Saline-Alkali Tolerant Rice, College of Coastal Agricultural Sciences, Guangdong Ocean University, Zhanjiang, China; ^2^ Key Laboratory for Quality Regulation of Tropical Horticultural Crops of Hainan Province, School of Horticulture, Hainan University, Haikou, China; ^3^ Xiangyang Academy of Agricultural Sciences, Xiangyang, China

**Keywords:** ammonium uptake, ammonium deficiency, ammonium transporter 2, gene expression, cassava

## Abstract

**Background:** Nitrogen (N), absorbed primarily as ammonium (NH_4_^+^) from soil by plant, is a necessary macronutrient in plant growth and development. Ammonium transporter (AMT) plays a vital role in the absorption and transport of ammonium (NH_4_^+^). Cassava (*Manihot esculenta* Crantz) has a strong adaptability to nitrogen deprivation. However, little is known about the functions of ammonium transporter AMT2 in cassava.

**Methods:** The cassava AMT2-type genes were identified and their characteristics were analyzed using bioinformatic techniques. The spatial expression patterns were analyzed based on the public RNA-seq data and their expression profiles under low ammonium treatment were studied using Real-time quantitative PCR (RT-qPCR) method. The cassava *AMT2* genes were transformed into yeast mutant strain TM31019b by PEG/LiAc method to investigate their functions.

**Results:** Seven AMT2-type genes (*MeAMT2.1-2.7*) were identified in cassava and they were distributed on 6 chromosomes and included two segmental duplication events (*MeAMT2.2/MeAMT2.4* and *MeAMT2.3/MeAMT2.5*). Based on their amino acid sequences, seven MeAMT2 were further divided into four subgroups, and each subgroup contained similar motif constitution and protein structure. Synteny analysis showed that two and four *MeAMT2* genes in cassava were collinear with those in the Arabidopsis and soybean genomes, respectively. Sixteen types of cis-elements were identified in the *MeAMT2* promoters, and they were related to light-, hormone-, stress-, and plant growth and development-responsive elements, respectively. Most of the *MeAMT2* genes displayed tissue-specific expression patterns according to the RNA-seq data, of them, three *MeAMT2* (*MeAMT2.3*, *MeAMT2.5*, and *MeATM2.6*) expressions were up-regulated under ammonium deficiency. Complementation experiments showed that yeast mutant strain TM31019b transformed with *MeAMT2.3*, *MeAMT2.5*, or *MeATM2.6* grew better than untransgenic yeast cells under ammonium deficiency, suggesting that MeAMT2.3, MeAMT2.5, and MeATM2.6 might be the main contributors in response to ammonium deficiency in cassava.

**Conclusion:** This study provides a basis for further study of nitrogen efficient utilization in cassava.

## Introduction

Plants utilize nutrients to maintain their normal growth and development. Nitrogen (N), mainly absorbed from soil, is an essential nutrient in growth and development of plants. N sources in soil include inorganic nitrogen (nitrate and ammonium), organic compounds and amino acids (Williams and Miller, 2001). Nitrate nitrogen and ammonium nitrogen are considered as main plant nitrogen sources. Lack of nitrogen will affect the synthesis of organic substances in plants, resulting in growth suppression, leaf yellowing and low yields (Tabata et al., 2014). When nitrogen is deficient, plants prefer absorbing ammonium nitrogen to nitrate nitrogen because absorption and assimilation of ammonium nitrogen consume less energy than that of nitrate nitrogen (Gazzarrini et al., 1999; Xuan et al., 2017). However, excessive ammonium is toxic to plants, therefore, its absorption and assimilation must be accurately regulated. The ammonium transport and homeostasis are controlled by unsaturated low-affinity absorption system (LATS, i.e., aquaporins or cation channels) and saturated high-affinity system (HATS, i.e., ammonium transporters) (Sohlenkamp et al., 2000). When the concentration of [NH_4_^+^]_ext_ is lower than 1 mM, HATS is mainly responsible for absorbing NH_4_^+^, and the absorption approaches Michaelis-Menten kinetics. Whereas when the concentration is higher than 1 mM, LATS is activated and plays a role in absorbing NH_4_^+^, with a linear unsaturated characteristic (Kronzucker et al., 1996; Wang et al., 1996).

Ammonium transporter (AMT) proteins, which are encoded by a small multigene family, play critical roles in regulating ammonium absorption and transport. There are 10 and six AMTs in rice and *Arabidopsis*, respectively (Gazzarrini et al., 1999; Sonoda et al., 2003). According to their sequences and phylogenetic relationships, AMT proteins in plant cells can be divided into two categories: AMT1 and AMT2 (Loqué and von Wirén, 2004). Many AMT1s have been reported in different plants (Ninnemann et al., 1994; Ranathunge et al., 2014; Li et al., 2015; Filiz and Akbudak, 2020; Yang et al., 2021), and it has been found that these AMT1 proteins function as high-affinity ammonium transporters (McDonald et al., 2012). Ammonium transport mediated by AMT1s is regulated by phosphorylation and dephosphorylation of the transporters (Loque et al., 2007; Neuhauser et al., 2007). It has recently been reported that CIPK23, a Ser/Thr kinase, can negatively impact the NH_4_^+^ transport activities of AtAMT1; 1 and AtAMT1; 2 by direct interactions with their C-termini (Straub et al., 2017). Compared with the studies on functional and regulation mechanisms of AMT1s, the reports for expression patterns and physiological functions of AMT2s are little, due to the large number of AMT2 family members and complex expression patterns (Sohlenkamp et al., 2002; Neuhäuser et al., 2009).

AMT2 ammonium transporters belong to the family of methylamine permease (MEP) as AMT1s. The primary structure of AMT2 proteins is quite different from that of AMT1 proteins, but their advanced structure is similar as that of AMT1 proteins (Howitt and Udvardi, 2000). AMT2s have a high transport capacity for NH_4_^+^ (Neuhäuser et al., 2009), so they play a main role in distribution and regulation of NH_4_^+^ between the above-ground and underground parts of plants (Sohlenkamp et al., 2000; Giehl et al., 2017). Evolutionary analysis showed the close correlation between AMT2 proteins and ammonium transporters in many thermophilic bacteria, which may be related to the horizontal transfer of MEP genes in prokaryotes (Howitt and Udvardi, 2000; Zheng et al., 2004; McDonald et al., 2012). *Arabidopsis thaliana* AMT2 family contains only one member (AtAMT2.1) (Yuan et al., 2007), while the AMT2 family in many other plants, including rice, can be divided into AMT2, AMT3, AMT4, AMT5 and other groups according to different clades in phylogenetic analysis (Li et al., 2009). AtAMT2.1 is a high-affinity ammonium transporter with a *Km* value of about 20 μM (Sohlenkamp et al., 2000; Sohlenkamp et al., 2002), but most of the AMT2 members are low-affinity ammonium transporters. Compared with *AMT1* genes that are mainly expressed in roots, AMT2 proteins distribute in different plant organs. For example, *AtAMT2.1* is widely expressed in roots, leaves, and other tissues of *Arabidopsis* plants, and play a critical role in the reuse of ammonium ions (Sohlenkamp et al., 2000). *CsAMT3.1* is a type of AMT2-type gene and has a high expression level in roots and green tissues, with the highest expression in mature leaves. Its function is related to transport and distribution of NH_4_^+^ in various tissues of tea plants (Zhang et al., 2018).

Cassava (*Manihot esculenta* Crantz) is an important raw material crop for food and energy (Ou et al., 2018). As a safe and nutritious high quality food, cassava has gradually been favored by more and more people. Cassava has a wide and strong adaptability and adverse resistance, especially to nitrogen deprivation (Xu et al., 2013). In our previous study, six cassava *AMT1* genes were identified, and MeAMT1; 1 was found to play a vital role in response to ammonium deficiency (Xia et al., 2022). However, little is known about the *MeAMT2* gene family. Herein, seven cassava *AMT2* genes (*MeAMT2.1-2.7*) were found from the cassava genome. The phylogeny, gene structures, genomic locations, and *cis*-elements, the expression patterns of these *MeAMT2* genes in different tissues and their expression profiles at low NH_4_^+^ level were investigated. Moreover, the functions of three *MeAMT2* genes (*MeAMT2.3*, *MeAMT2.5*, and *MeATM2.6*) were explored using a yeast complementation system. The present results may provide valuable information for further studies on AMT2 functions and nitrogen efficient utilization in cassava.

## Materials and methods

### Plant materials and treatments

The plant material of cassava cultivar ‘SC8’ (*Manihot esculenta* Crantz cv. SC8) was grown in a greenhouse with conditions: temperature, 28°C; light intensity, 300 μmol m^−2^·s^−1^; relative humidity, 60%, light/darkness, 16 h/8 h (Xia et al., 2022). The seedlings were subjected to a 1-week culture in Afdaling nutrient solution (Hu et al., 2016), followed by low ammonium treatment in medium containing 0.05 mM NH_4_Cl for 6, 12, 24 and 48 h, and the leaves, roots, and stems were collected at each time point, all samples were frozen in liquid nitrogen and stored at −80°C for further use.

### Identification and characterization of AMT2 family members in cassava

To identify the AMT2 transporters in cassava, the AMT2 protein sequences from *A. thaliana* (Sohlenkamp et al., 2000) were utilized as query sequences with E-value cutoff set as 1e-5 to perform a local BLASTP against the whole-genome data of cassava (*Manihot esculenta* v6.1) (phytozome v11.0, https://phytozome.jgi.doe.gov/pz/portal.html). The gene location information was obtained. The theoretical isoelectric point (pI) and molecular weight (MW) of the MeAMT2 proteins were obtained using the ExPASy online software (http://web.expasy.org/protparam/). PSORT instrument (https://www.genscript.com/) was used to characterize the subcellular localization of MeAMT2 proteins.

### Analysis of phylogenetic, conserved motifs and gene structure

In order to understand the phylogenetic relationships of AMT2 proteins in different plant species, the sequences of AMT2 proteins from *A. thaliana* (Gazzarrini et al., 1999), rice (*Oryza sativa*) (Sonoda et al., 2003), soybean (*Glycine max*) (Kobae et al., 2010), maize (*Zea mays*) (Hui et al., 2022), and *Lotus japonicus* (Wang et al., 2022) were obtained from the previous study, and the sequences from *Ricinus communis,* belonging to Euphorbiaceae, were identified using the identification method for the cassava AMT2 gene family. Multiple sequence alignment using MUSCLE and default parameters was applied to evaluate the evolutionary relationship of MeAMT2s proteins in cassava (Edgar, 2004), and a neighbor-joining (NJ) phylogenetic tree was established based on the alignment utilizing the MEGA version X software (Kumar et al., 2018) with 1000 bootstrap replications. The online software Multi Em for Motif Elicitation (MEME Suite 4.12.0) (http://meme-suite.org/tools/meme) was used to analyze the conserved motifs of MeAMT2 proteins with optimization parameters: maximum number of motifs, 10; optimum width, 6–50; number of repetitions, any; as well as other default parameters. The *MeAMT2* gene structures were analyzed using Gene Structure Display Server (GSDS) (http://gsds.cbi.pku.edu.cn/).

### Analysis of gene collinearity and chromosomal distribution

The *MeAMT2* genes were mapped to the chromosomes utilizing TBtools software (Chen et al., 2020) according to the information obtained from the cassava genome database. Simultaneously, gene duplication of *MeAMT2* genes was analyzed utilizing MCScanX software (Wang et al., 2012) and illustrated with TBtools. The nucleotide substitution parameters *Ks* (synonymous) and *Ka* (non-synonymous) of the duplicated genes were assessed using TBtools, and then the *Ka*/*Ks* ratio was calculated. In addition, the gene duplication information from cassava, soybean, and *A. thaliana* was analyzed using MCscanX software, followed by integral visualization of synteny with TBtools software (Wang et al., 2012; Chen et al., 2020).

### Analysis of *cis*-elements in *MeAMT2* promoters

The 2.0-kb upstream sequences of the *MeAMT2* genes were acted as the promoters and submitted to PlantCARE (http://bioinformatics.psb.ugent.be/webtools/plantcare/html/). The *cis*-regulatory elements were identified and visualized by TBtools.

### Spatial expression profiles of *MeAMT2s* based on RNA-seq data

The Transcripts Per Million (TPM) values of the *MeAMT2* genes obtained from the RNA-seq data in the SRA dataset (https://www.ncbi.nlm.nih.gov/sra) (SRP076160) (Wilson et al., 2017) were utilized to examine the expression profiles of *MeAMT2* genes in 11 selected cassava tissues including leaf, root, petiole, stem, midevein, fibrous root, lateral bud, middle storage root, early storage root, storage root and last storage root. The heatmap of *MeAMT2* genes was constructed by TBtools.

### Real-time quantitative PCR (RT-qPCR) analysis of *MeAMT2* genes

The total RNA of cassava roots, stems and leaves was extracted according to the instructions of the Plant Total RNA Extraction Kit (TIANGEN, China, DP437). The first-strand cDNA was synthesized using the FastKing cDNA First Strand Synthesis Kit (TIANGEN, China, KR116-02). The transcript levels of *MeAMT2* genes were analyzed using RT-qPCR on an ABI 7900HT RT-PCR system (TaKaRa, Japan) using SuperReal PreMix Plus (SYBR Green I) fluorescence quantification kit (TIANGEN, China, FP205-02) with *Actin* gene as internal reference control (Mo et al., 2018). The PCR amplification conditions were referred to the previous study (Mo et al., 2018). The experiments were repeated three times, and the relative expression of target genes was determined using the 2^−ΔΔCt^ method (Livak and Schmittgen, 2001), where ΔΔCt = (Ct_target gene_—Ct_internal reference gene_)_experimental group_—(Ct_target gene_—Ct_internal reference gene_)_control group_. Three independent biological replicates were conducted. The primers used are designed using Primer Premier five software and shown in Supplementary Table S1.

### Construction of yeast expression vector and functional analysis

To investigate the functions of the *MeAMT2* genes, these genes were cloned using the cDNA as templates and inserted into the yeast expression vector p416. The restriction sites for construction could be seen in the primer sequences. After digestion and sequencing, the recombinant plasmids were confirmed. The plasmids p416 or p416-*MeAMT2s* were then transformed into the *Saccharomyces cerevisiae* mutant strain TM31019b (MATα ura3 mep1Δ mep2Δ:LEU2 mep3Δ:KanMX2) (Marini et al., 1997) using the PEG/LiAc method (Yin et al., 2020). The TM31019b strains were then grown on a SD/-ura solid medium to obtain positive clones (Li et al., 2017). The successful transformants were then grown on yeast nitrogen base medium containing 1 mM arginine (control) or low ammonium (different concentrations of NH_4_Cl) for 3–5 days at 30°C. The primers used are designed using Primer Premier five software and shown in Supplementary Table S1.

## Results

### Identification of the *AMT2* gene family in cassava

**FIGURE 1:**
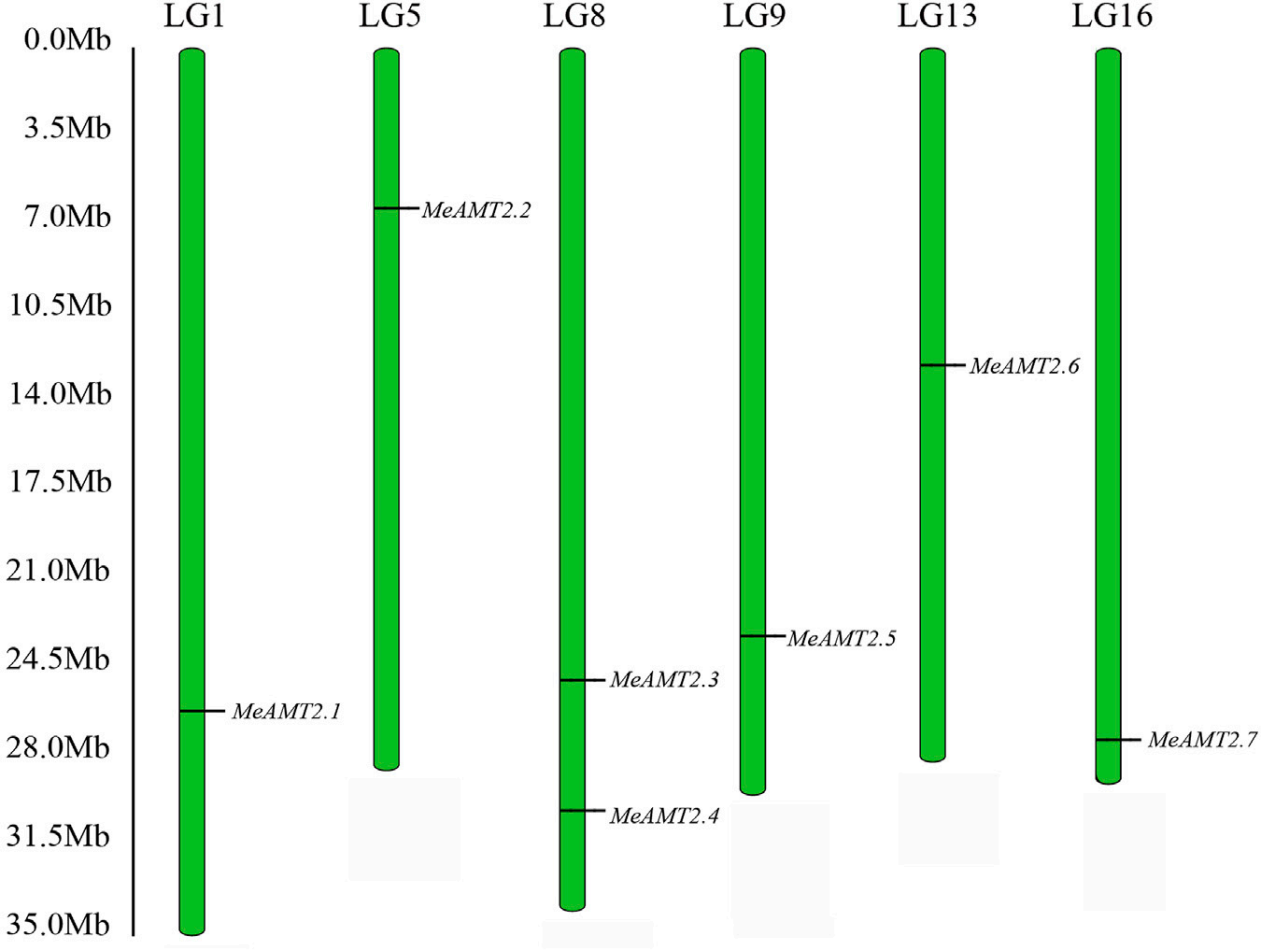
Chromosomal distribution of *MeAMT2* genes. The chromosome number is listed above each chromosome while the numbers on the left represent the locations of the *MeAMT2* genes.

Through a BLASTP search using *Arabidopsis* AMT2 protein, seven *AMT2* genes were identified in the cassava genome. These seven *MeAMT2* genes distributed on six chromosomes (LG1, LG5, LG8, LG9, LG13, and LG16) were named *MeAMT2.1* to *MeAMT2.7* accordingly (Figure 1; Table 1). The physicochemical characteristics of the *MeAMT2* genes are summarized in Table 1, with coding sequences (CDSs) ranging from 1272 (*MeAMT2.5*) to 1473 (*MeAMT2.6*) bp, and the length, MW and pI of the predicted proteins ranging from 423 (MeAMT2.5) to 490 (MeAMT2.6) amino acids, from 46.39 (MeAMT2.5) to 52.72 (MeAMT2.6) kDa, and from 5.99 (MeAMT2.4) to 9.29 (MeAMT2.5), average of 7.05, respectively. Furthermore, all the proteins were predicted to be located in cell membrane.

**TABLE 1:** Physicochemical properties of MeAMT2 proteins.

Gene name	Locus gene	Chromosome location	ORF length (bp)	Amino acid number (aa)	Molecular weight (kDa)	Isoelectric point	Predicted location
MeAMT2.1	OAY60943	LG1: 26124998.26126863	1431	476	52.40	6.30	Cell membrane
MeAMT2.2	OAY49780	LG5: 6293419.6295092	1293	430	46.70	8.14	Cell membrane
MeAMT2.3	OAY43751	LG8: 24909063.24914634	1461	486	52.19	6.65	Cell membrane
MeAMT2.4	OAY44236	LG8: 30051650.30053670	1425	474	51.69	5.99	Cell membrane
MeAMT2.5	OAY41524	LG9: 23165592.23166972	1272	423	46.39	9.29	Cell membrane
MeAMT2.6	OAY33230	LG13:12474468.12477951	1473	490	52.72	6.54	Cell membrane
MeAMT2.7	OAY27357	LG16: 27264011.27265788	1458	485	52.51	6.45	Cell membrane

### Phylogenetic analysis of cassava AMT2 proteins

**FIGURE 2:**
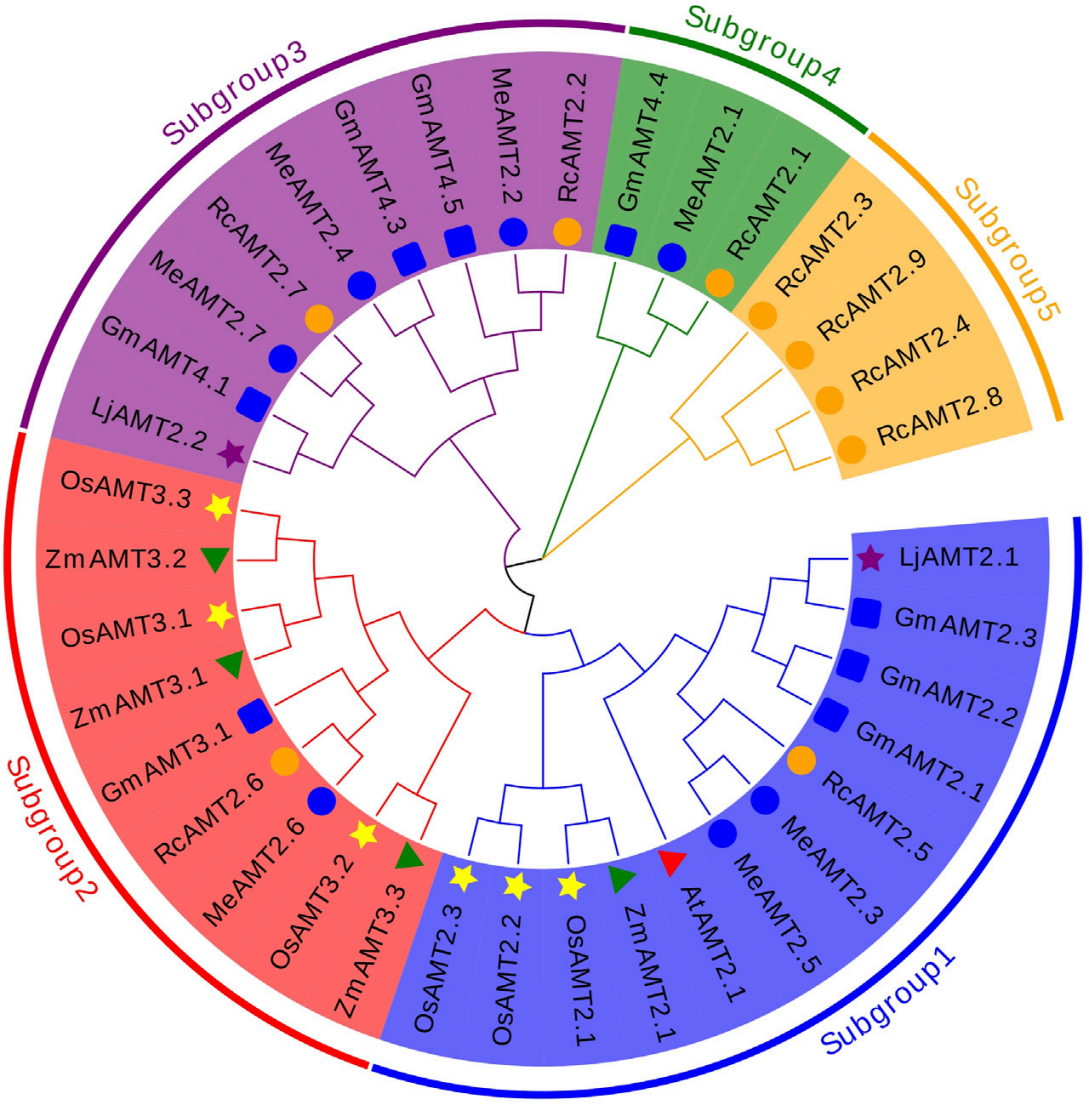
Phylogenetic analyses of AMT2 proteins. The phylogenetic tree of the AMT2 proteins was constructed using MEGA-X software with default parameters. The five subgroups are marked with different colors. The blue circles represent MeAMT2s, The orange circles represent RcAMT2s, the red triangles represent AtAMT2s, the yellow stars represent OsAMT2s, the purple stars represent LjAMT2s, the green triangles represent ZmAMT2s, and the blue squares represent GmAMT2s.

The evolutionary relationship between cassava AMT2 proteins and those from other plant species was explored next. A total of 37 protein sequences from cassava, *R. communis*, *G. max*, *L. japonicus*, *Z. mays*, *O. sativa*, and *A. thaliana* were used to construct a NJ phylogenetic tree. These proteins were clustered into five subgroups: subgroup 1 consisted of two MeAMT2s (MeAMT2.3 and MeAMT2.5), one RcAMT2 (RcAMT2.5), three GmAMT2s (GmAMT2.1, GmAMT2.2 and GmAMT2.3), three OsAMT2s (OsAMT2.1, OsAMT2.2 and OsAMT2.3), one LjAMT2 (LjAMT2.1), one ZmAMT2 (ZmAMT2.1), and one AtAMT2 (AtAMT2.1); subgroup 2 consisted of one MeAMT2 (MeAMT2.6), one RcAMT2 (RcAMT2.6), one GmAMT2 (GmAMT3.1), three OsAMT2s (OsAMT3.1, OsAMT3.2 and OsAMT3.3), and three ZmAMT2s (ZmAMT3.1, ZmAMT3.2 and ZmAMT3.3); subgroup 3 consisted of three MeAMT2s (MeAMT2.2, MeAMT2.4 and MeAMT2.7), two RcAMT2s (RcAMT2.2 and RcAMT2.7), three GmAMT2s (GmAMT4.1, GmAMT4.3 and GmAMT4.5), and one LjAMT2 (LjAMT2.2); subgroup 4 contained one MeAMT2 (MeAMT2), one RcAMT2 (RcAMT2.1), and one GmAMT2 (GmAMT4.4); subgroup 5 contained four RcAMT2s .1 (RcAMT2.3, RcAMT2.4, RcAMT2.8 and RcAMT2.9). The Euphorbiaceae crops, cassava and *R. communis*, had similar distribution in subgroups 1 to 4. Differently, only *R. communis* AMT2 proteins were present in subgroup 5 (Figure 2).

### Analysis of conserved motifs and gene structure of the cassava *AMT2* genes

**FIGURE 3:**
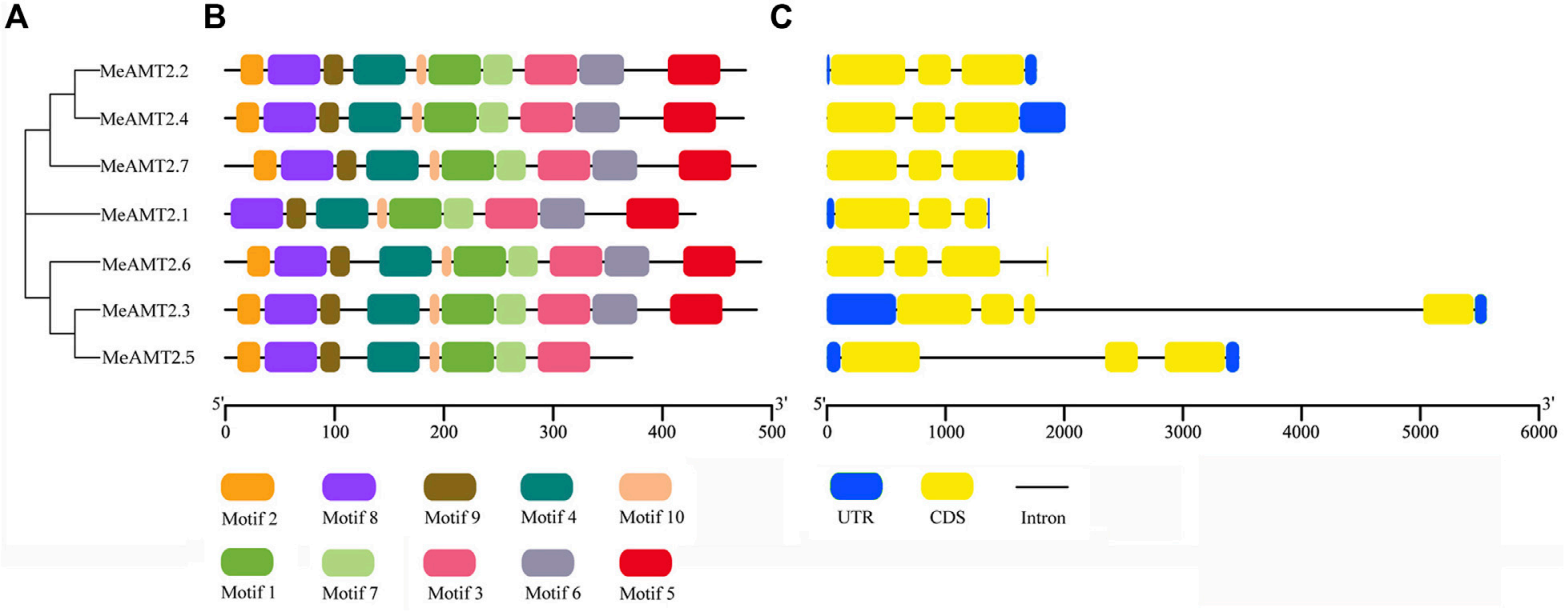
Motif compositions and gene structures of *MeAMT2s* **(A)** Phylogenetic analyses of MeAMT2 proteins **(B)** Distribution of conserved motifs in MeAMT2 proteins. The different colored boxes represent different motifs and their positions in each MeNRT2 protein sequence. Scale bar indicates number of amino acids **(C)** Exon-intron structures of *MeAMT2* genes. The introns and exons are shown with black lines and yellow boxes, respectively. Green boxes represent the untranslated regions. Scale bar indicates number of nucleic acids (bp).

To further characterize structural features of the *AMT2* genes, the gene structures and distribution of conserved motifs were analyzed using the MEME program. As shown in Figure 3B, 10 putative conserved motifs (named motifs 1–10) were detected in most of the MeAMT2 protein sequences with length ranging from 11 to 50 amino acid residues (Supplementary Figure S1). All the MeAMT2 proteins except MeAMT2.1 (lacking motif two in N-terminal region) and MeAMT2.5 (lacking motif five and six in C-terminal region) contained the 10 motifs (Figure 3B). The structure of the *MeAMT2* genes contained three exons separated by two introns except for *MeAMT2.3* and *MeAMT2.6* (Figure 3C), which contained four exons and three introns. Moreover, most of the *MeAMT2* genes in the same subgroups shared similar gene structures and motif distributions (Figure 3).

### Analysis of gene duplication and synteny of *MeAMT2s*

**FIGURE 4:**
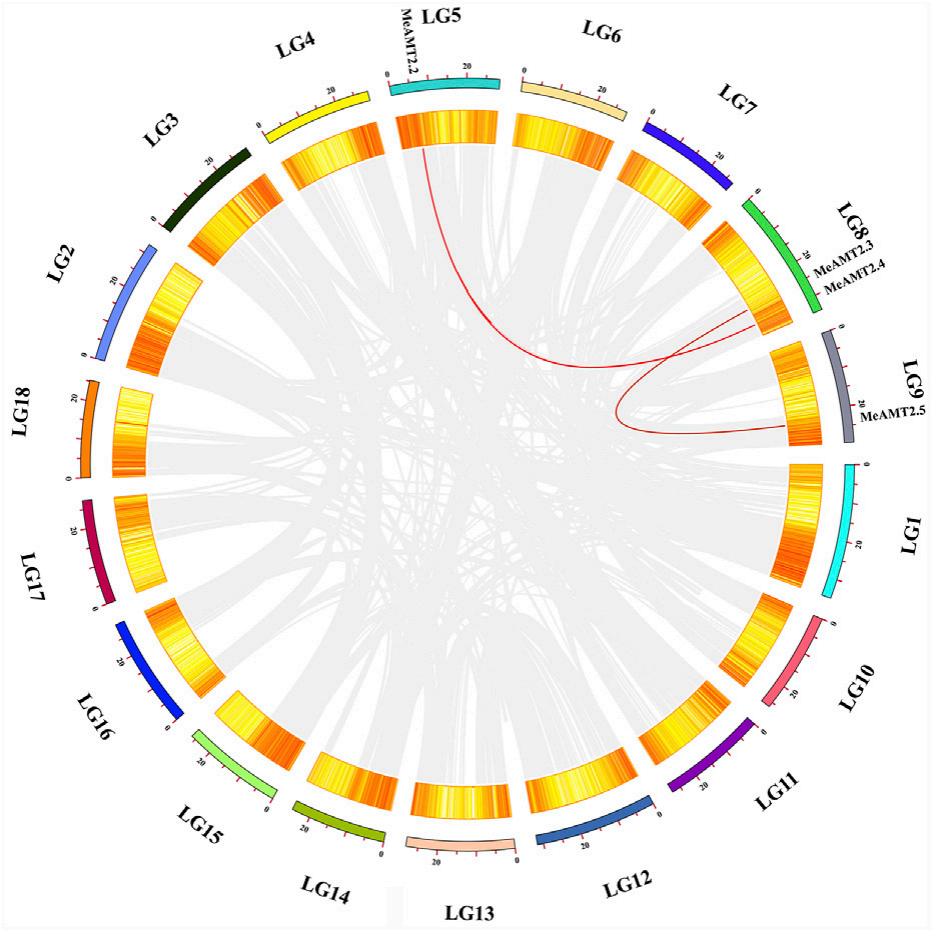
Schematic representations of the chromosomal distribution and interchromosomal relationships among cassava *AMT2* genes. Chromosomes are represented in different colors with the chromosomal number indicated below each chromosome. Gray lines indicate synteny blocks within the cassava genome and red lines between *MeAMT2* genes represent segmental duplication events that occurred in the cassava *AMT2* gene family.

Figure 4 shows the gene duplication events. It can be seen that the *MeAMT2* members were unevenly distributed in six among the 18 cassava chromosomes with only segmental duplication but no tandem duplication genes were detected. Two pairs of observed genes, *MeAMT2.2*/*MeAMT2.4* and *MeAMT2.3*/*MeAMT2.5*, were regarded as segmental duplication genes (Figure 4), suggesting the critical roles of segmental duplication in the expansion of *MeAMT2* family. The value of *Ka*/*Ks* can reflect the pressure of selection for a gene during evolution. The *Ka*/*Ks* values of the duplicated genes were both less than 1, suggesting that these *MeAMT2* genes were influenced by purifying selection during evolution. The estimated time of duplication for paralogous genes indicated that all paralogs were ancient (from 16.4 to 56.6 Mya) (Table 2).

**TABLE 2:** *Ka*/*Ks* values of *MeAMT2* duplicated genes.

Gene name_1	Gene name_2	Ka	Ks	Ka/Ks	Data (Mya)^a^
MeAMT2.2	MeAMT2.4	0.149067428	1.698264985	0.087776306	56.6
MeAMT2.3	MeAMT2.5	0.08511017	0.492306113	0.172880588	16.4

^a^ The divergence time was estimated according to formula: T = Ks/2λ. The clock like rate λ) was 1.5 × 10^–8^ substitutions per site per year (Lynch and Conery, 2000). Mya: million years ago.

**FIGURE 5:**
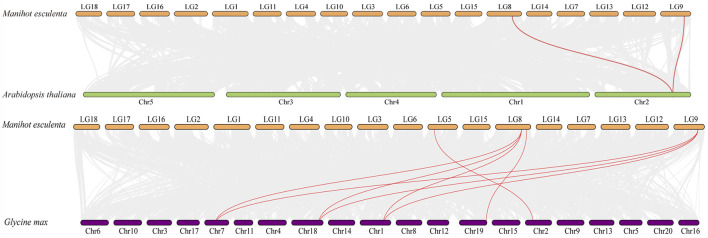
Synteny analyses of *AMT2* genes between cassava, *Arabidopsis* and soybean. Gray lines in the background indicate the collinear blocks within cassava and other plant genomes, while the red lines highlight the syntenic *AMT2* gene pairs.

The comparative syntenic map was then constructed to show the associations between cassava and *Arabidopsis*, rice and soybean. As shown in Figure 5, four *MeAMT2* genes showed a syntenic correlation with genes in soybean, and two *MeAMT2* genes exhibited a syntenic correlation with genes in *Arabidopsis*, while no associations were found between *MeAMT2s* and *OsAMT2s* (data not shown). *MeAMT2.3* and *MeAMT2.5* were found to have syntenic relationships with *AtAMT2.1*, *MeAMT2.2* had syntenic relationship with *GmAMT3.1*, *MeAMT2.3* had syntenic relationships with *GmAMT2.1*, *GmAMT2.2*, and *GmAMT2.3*, *MeAMT2.4* had syntenic relationship with *GmAMT4.3*, and *MeAMT2.5* had syntenic relationships with *GmAMT2.1*, *GmAMT2.2*, and *GmAMT2.3* (Figure 5, Supplementary Table S2).

### Analysis of *cis*-elements in the promoter regions of *MeAMT2* genes

**FIGURE 6:**
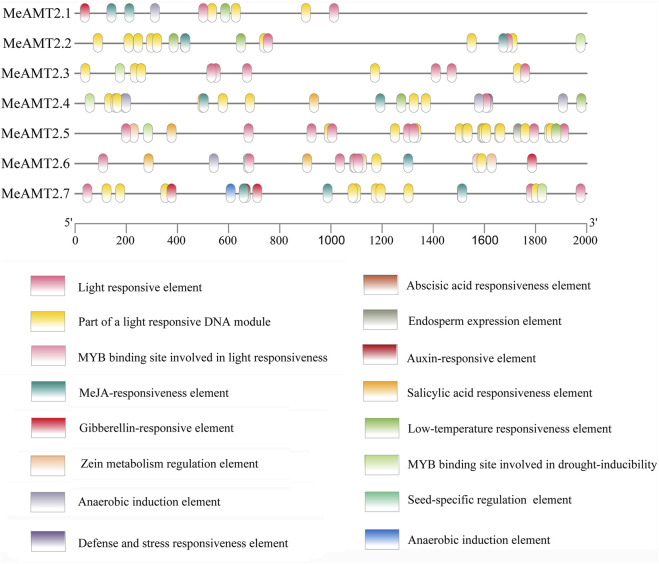
Analysis of *cis*-elements in the promoter regions of *MeAMT2* genes. The potential elements in the promoter regions 2-kb upstream of the *MeAMT2* genes were analyzed by PlantCARE. The upstream length to the translation start site can be inferred according to the scale at the bottom. Different color boxes represent different functions as described.

The 2000 bp upstream sequences of start codons of the *MeAMT2* genes were exacted as promoter sequences and used to analyze their *cis*-acting elements with PlantCARE software. Based on their biological functions, these 16 types of *cis*-acting elements were classified into four categories: 1) the light-responsive elements, the most numerous categories. The *MeAMT2.1* and *MeAMT2.5* promoter regions had the smallest and largest number of light-responsive elements, respectively (5, 23). 2) The hormone response related elements, including salicylic acid responsiveness element (SARE), abscisic acid responsiveness element (ABRE), gibberellin-responsive element, MeJA-responsiveness element, and auxin-responsive element (ARE). The ABRE only existed in the *MeAMT2.5* and *MeAMT2.6* promoter regions, indicating these two genes may be involved in ABA signaling pathway. The ARE only existed in the *MeAMT2.7* promoter region, indicating this gene may be involved in auxin signaling pathway. The MeJA-responsiveness element existed in most promoter regions except for *MeAMT2.3* and *MeAMT2.5*.3) The *cis*-acting elements associated with plant development. For example, the *MeAMT2.6* promoter contained seed-specific regulation elements. 4) The stress-related element, which was uncommon in the *MeAMT2* promoter region. The promoter region of MeAMT2.4 had a defense and stress responsiveness element, and the drought-inducibility element existed in the promoter regions of *MeAMT2.2*, *MeAMT2.3*, *MeAMT2.4*, *MeAMT2.5* and *MeAMT2.*7. The low-temperature responsiveness element (LTR) existed in the promoter regions of *MeAMT2.1*, *MeAMT2.2*, *MeAMT2.4* and *MeAMT2.5* (Figure 6).

### Expression profiles of *AMT2* genes in different tissues of cassava based on RNA-seq

**FIGURE 7:**
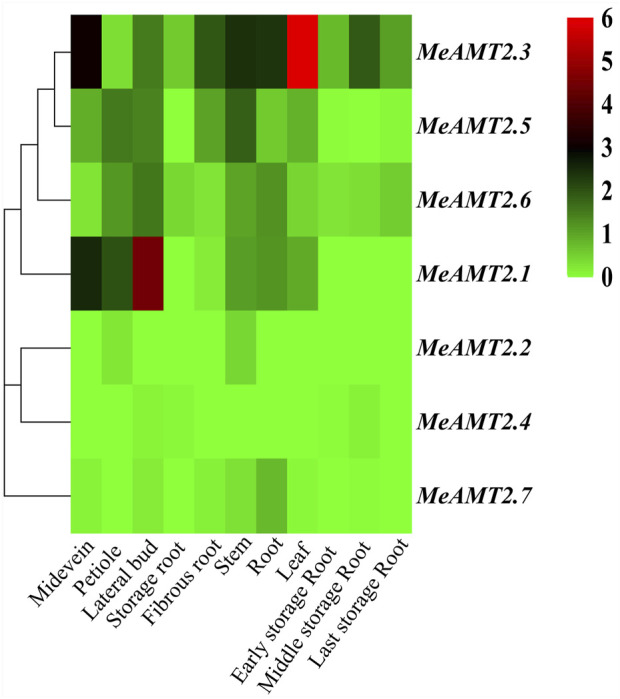
Hierachical clustering of *MeAMT2* gene expression profiles across different cassava tissues. Data were normalized relative to each gene’s mean expression value across all tissues and log2-transformed. TPM (transcripts per kilobase million) values were used to create heat map showing the expression of *MeAMT2* genes in different tissues. The expression level ranges from low expression (green) to high expression (red).

The spatial expression profiles of *MeAMT2* genes in 11 cassava tissues including root, leaf, stem, storage root, fibrous root, midvein, petiole, lateral buds, early, medium and late storage roots were analyzed using RNA-seq data (SRP076160) of cassava from the public SRA database (Wilson et al., 2017) (Figure 7, Supplementary Table S3). The three genes, *MeAMT2.3*, *MeAMT2.5* and *MeAMT2.6*, exhibited higher expression than other genes in most of the tested tissues. *MeAMT2.3*, *MeAMT2.5* and *MeAMT2.6* were highly expressed in leaves, lateral buds, and stems, respectively. *MeAMT2.1* gene was also expressed in all tissues with a much lower level than the above three genes. The remaining three genes (*MeAMT2.2*, *MeAMT2.4* and *MeAMT2.7*) were only expressed in some tissues at low levels.

### Expression patterns of cassava *AMT2* genes under low ammonium conditions

**FIGURE 8:**
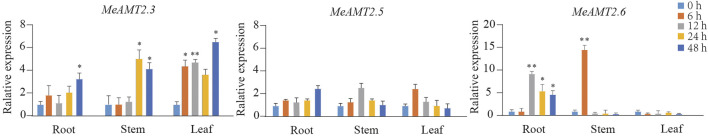
Expression profiles of *MeAMT2* genes under ammonium deficiency. Data were analyzed by real-time quantitative PCR (RT-qPCR) using *Actin* gene as an internal cotrol. The unstressed level (0 h) was regarded as a standard. Values are the mean ± SE, *n* = 3. Vertical bars indicate the standard deviation. *p < 0.05 and **p < 0.01.

To access whether ammonium deficiency influences the expression of *MeAMT2* genes, we treated cassava seedlings with 0.05 mM NH_4_Cl for different periods. The results of RNA detection (Supplementary Figure S2A) and cDNA amplification (Supplementary Figure S2B) showed that the qualities were good for subsequent analysis. The three *MeAMT2* genes (*MeAMT2.3*, *MeAMT2.5* and *MeAMT2.6*) with higher spatial expression levels were selected for analysis in roots, stems and leaves under low ammonium conditions using RT-qPCR. Results showed that these three genes were up-regulated in different degrees under the condition of ammonium deficiency (Figure 8). The expression of *MeAMT2.3* in leaves, roots, and stems was similarly slightly up-regulated after low ammonium treatment. The maximum expression level of *MeAMT2.3* in roots, stems and leaves was reached at 48, 24 and 48 h after low ammonium treatment, respectively, which was about 3.10, 4.79, and 5.95 times of that at 0 h, respectively. The maximum expression level of *MeAMT2.5* in roots, stems and leaves was reached at 48, 12 and 6 h after low ammonium treatment, respectively, which was about 2.60, 2.58, and 2.57 times of that at 0 h, respectively. The expression level of *MeAMT2.6* in leaves did not change significantly after low ammonium treatment, but it was obviously up-regulated in roots and stems. *MeAMT2.6* reached the highest expression level in roots and stems at 12 and 6 h after low ammonium treatment, which was about 10.59 and 15 times of that at 0 h, respectively.

### Functional analysis of MeAMT2s in transgenic yeast

**FIGURE 9:**
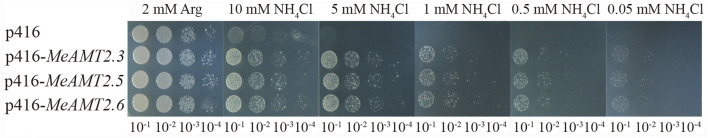
The effect of low ammonium treatment on yeast growth. p416-*MeAMT2.3*, p416-*MeAMT2.5*, p416-*MeAMT2.6*, and p416 were transformed into the TM31019b mutant yeast strain, respectively. The transgenic yeast cells were pre-cultured to saturation, and serial tenfold dilutions of the yeast cells were spotted on SD/-ura plates as previously described. After 3–5 days, the growth of the yeast cells on the plates was recorded. SD/-ura supplemented with 2 mM Arg was used as a control.

The three genes (*MeAMT2.3*, *MeAMT2.5* and *MeAMT2.6*) were cloned (Supplementary Figure S3A) and inserted into the yeast expression vector p416 (Supplementary Figure S3B) and then transformed into TM31019b mutant strain to further investigate the functions of *MeAMT2* genes in response to low ammonium. Results showed that the growth of yeast cells transformed with either empty vector or *MeAMT2* genes was similar under normal condition with 2 mM Arg. However, yeast cells transformed with empty vector p416 grew poorly with decreasing concentration of ammonium, and could not grow on SD/-ura medium containing 5 mM NH_4_Cl. In contrast, the *MeAMT2.3*-, *MeAMT2.5*-, or *MeAMT2.6*-transgenic TM31019b still grew well with decreasing concentration of NH_4_Cl, and could grow even under 0.05 mM NH_4_Cl treatment (Figure 9).

## Discussion

Nitrogen is a plant-demanded mineral element necessary for plant growth (Williams and Miller, 2001). Nitrogen plays an extremely important role in crop growth, development, yield and quality. However, excessive nitrogen fertilizer application is not only a waste, but can also negatively affect crop growth and cause serious environmental pollution (Liu et al., 2013; Zhang et al., 2016). Plants can absorb both organic nitrogen (including peptides, amino acids, *etc.*) and inorganic nitrogen (including ammonium and nitrate nitrogen) from soil with nitrate nitrogen (NO_3_^−^) and ammonium nitrogen (NH_4_^+^) as the main forms (Xu et al., 2012). In agricultural production, regardless of soil type, the application of nitrogen fertilizer will lead to NH_4_^+^ becoming the main form in a short period of time (Xu and Takahashi, 2020). Since NH_4_^+^ consumes less energy than NO_3_^−^ in the process of assimilation and utilization, NH_4_^+^ is considered the dominant nitrogen source (Socci and Templer, 2011). However, excessive absorption of NH_4_^+^ can result in toxic effects on crops, inducing leaf yellowing and growth inhibition. Therefore, maintaining an appropriate range of NH_4_^+^ absorption from the soil is critical for plant growth.

Plants absorb and transport NH_4_^+^ mainly through ammonium transporters (Yuan et al., 2007; Hao et al., 2020). The AMT gene family has been divided into two subfamilies: AMT1 subfamily (AMT1 cluster) and AMT2 subfamily (AMT2/3/4 cluster) (Koegel et al., 2013; Hao et al., 2020). At present, the AMT family genes in *Arabidopsis* have been well studied. The AtAMT family has been divided into two subfamilies: AtAMT1 and AtAMT2. The AtAMT1 subfamily contains five members: AtAMT1; 1∼AtAMT1; 5, and the AtAMT2 subfamily contains only AtAMT2; 1 (Gazzarrini et al., 1999). Cassava, as a tropical economic crop, is highly adaptable and can achieve high yield even in arid and barren mountainous areas. However, few studies have been on nitrogen utilization of cassava thus far. We have identified the cassava *NRT2* and *AMT1* gene families, and studied their response to low nitrogen stress, and found that MeNRT2.2 and MeAMT1; 1 could improve the plant growth of transgenic *A. thaliana* under low nitrate or ammonium stress (Xia et al., 2022; You et al., 2022). Our results suggest that these two genes play important roles in cassava responding to low nitrogen stress. In this study, we identified seven *AMT2* genes from cassava genome, similar to those of rice (at least seven *OsAMT2* members) (Suenaga et al., 2003), but more than that of *Arabidopsis* (only one *AtAMT2* member) (Sohlenkamp et al., 2002), indicative of a possible gene gain event in the evolutionary process.

Phylogenetic analysis showed that the seven MeAMT2 and AMT2 proteins from *Ricinus communis*, *Lotus japonicus,* rice, soybean, maize and *Arabidopsis* could be divided into five subgroups (Figure 2). Cassava AMT2 is closely correlated with *Ricinus communis* AMT2 protein, both of the plants belonging to Euphorbiaceae. For example, MeAMT2.3 and MeAMT2.5 are highly similar to RcAMT2.5, MeAMT2.1/2.2/2.6/2.7 are highly similar to RcAMT2.1/2.2/2.6/2.7, respectively (Figure 2). In addition, the MeAMT2.4 was found to be closed to soybean GmAMT4.3. Synteny analysis also showed that *MeAMT2.4* was correlated with *GmAMT4.3* (Figure 5, Supplementary Table S2). Furthermore, *MeAMT2.3* and *MeAMT2.5* were found to be correlated with multiple genes from *Arabidopsis* and *G. max*, both of which were correlated with *AtAMT2.1*, *GmAMT2.1*, *GmAMT2.2* and *GmAMT2.3* (Figure 5, Supplementary Table S2), suggesting that MeAMT2.3 and MeAMT2.5 might have multiple functions. However, we did not find collinear pairs between monocot rice and cassava (data not shown), suggesting these orthologous pairs might be formed after differentiation of dicot and monocot plants (You et al., 2022). Analysis of conserved motifs showed that almost all MeAMT2 proteins, except MeAMT2.1 and MeAMT2.5, contained these ten motifs in a consistent order. The difference is that MeAMT2.1 lacked motif two in the N-terminus, while MeAMT2.5 lacked motif five and six in the C-terminus, suggesting loss of C- or N-terminus might have happened in some MeAMT2 members during evolution (Liu et al., 2018). Except for *MeAMT2.3* and *MeAMT2.6*, which contained four exons and three introns, most of the *MeAMT2* genes contained three exons and two introns (Figure 3). Similarly, three exons were found in *Pyrus betulaefolia* (Li et al., 2016), and five exons were found in Chinese cabbage *AMT2* genes (Zhu et al., 2018). Moreover, we found segmental duplicated genes (*MeAMT2.2*/*MeAMT2.4*, *MeAMT2.3*/*MeAMT2.5*) in synteny analysis (Figure 4). The intron loss rate has been shown to be faster than the intron gain rate after segmental duplication (Nuruzzaman et al., 2008). Based on this, *MeAMT2.5* (containing two introns) may be younger than *MeAMT2.3* (containing three introns) (Figure 3C; Figure 4), which might be diverged at 16.4 Mya (Table 2). In contrast, the cassava *AMT1* genes contain fewer exons; only *MeAMT1;2* gene contains three exons, and all the other *MeAMT1* genes contain no introns (Xia et al., 2022). This indicates that there are differences in evolution between *AMT1* and *AMT2* gene families in cassava, and *AMT2* gene family may have more intron insertion during evolution.

In addition, various light responsive, hormone responsive (e.g., ARE, ABRE, SARE, *etc.*), stress-responsive (drought-responsive and LTR) and plant growth and development-related elements were found in the *MeAMT2* promoter regions (Figure 6). But different *cis*-acting elements were found in each *MeAMT2* promoter, indicating that cassava *MeAMT2* genes exert multiple or specific functions. Previous studies showed that besides contributing to absorption of ammonium in roots, *AtAMT2;1* is mainly responsible for transport of ammonium from roots to shoots (Sohlenkamp et al., 2000; Giehl et al., 2017). The expression level of *OsAMT3.1* is relatively low, while *OsAMT2.1* is constitutively expressed in both shoots and roots and is not regulated by nitrogen sources (Suenaga et al., 2003). Our study observed tissue specificity for the expression of *MeAMT2* genes in cassava (Figure 7), indicating they might have different functions. *GmAMT4.1* showed specific expression in arbusculated cortical cells and localized on the periarbuscular membrane (Kobae et al., 2010). The *LjAMT2.2* gene was significantly upregulated when inoculating with arbuscular mycorrhizal fungi (AMF) (Wang et al., 2022). Phylogenetic relationship analysis showed that MeAMT2.2, MeAMT2.4, MeAMT2.7, GmAMT4.1, and LjAMT2.2 were all assigned to subgroup 3 (Figure 2). These results suggested that these cassava AMT2 proteins might be responsible for ammonium uptake in arbuscular mycorrhiza symbiosis. In this study, we selected *MeAMT2.3*, *MeAMT2.5* and *MeAMT2.6* for further analysis due to higher spatial expression levels. The expression patterns of the three genes were similar under low ammonium stress, especially the *MeAMT2.6* gene, which exhibited a significant up-regulation in stems and roots (Figure 8). The function investigation showed that MeAMT2.3, MeAMT2.5 and MeAMT2.6 all could enhance the growth of transgenic yeast under low ammonium condition (Figure 9), suggesting that they might be involved in response to ammonium deficiency.

## Conclusion

In conclusion, seven *MeAMT2* genes were identified from cassava, and the corresponding proteins were divided into four subgroups based on the phylogeny with other plant species. The gene location on chromosomes, physicochemical property, conserved motifs and gene structures were further analyzed. Genes in the same subgroup exhibited similar structures and properties. Two pairs of segmental duplicated genes were identified in the *MeAMT2* family. Spatial expression analysis showed tissue-specific expression for these genes. RT-qPCR analysis showed up-regulation of *MeAMT2.3*, *MeAMT2.5*, and *MeAMT2.6* genes under ammonium deficiency. Yeast strains overexpressing the above genes grew well under ammonium deficiency. This study provides substantial valuable reference information for further study of nitrogen efficient utilization in cassava.

## Data Availability

The datasets presented in this study can be found in online repositories. The names of the repository/repositories and accession number(s) can be found in the article/Supplementary Material.
