# Supplementary figures and images for: Genome-wide identification, expression profiling, and functional analysis of ammonium transporter 2 (AMT2) gene family in cassava (Manihot esculenta crantz)
Source: Front Genet. 2023 Feb 22;14:1145735. doi: 10.3389/fgene.2023.1145735 (PMC9992417; doi:10.3389/fgene.2023.1145735)

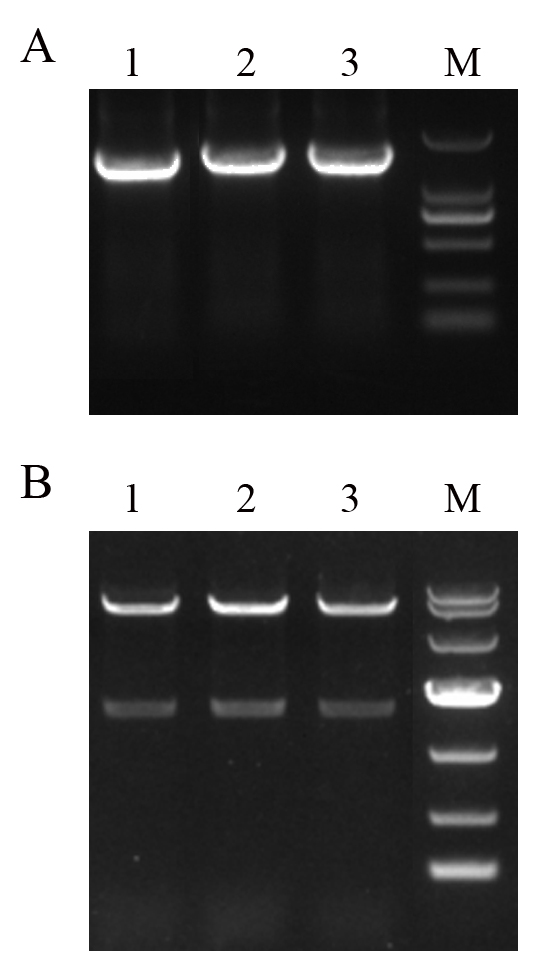

Supplement: Supplementary file 2 [file Image3.JPEG]

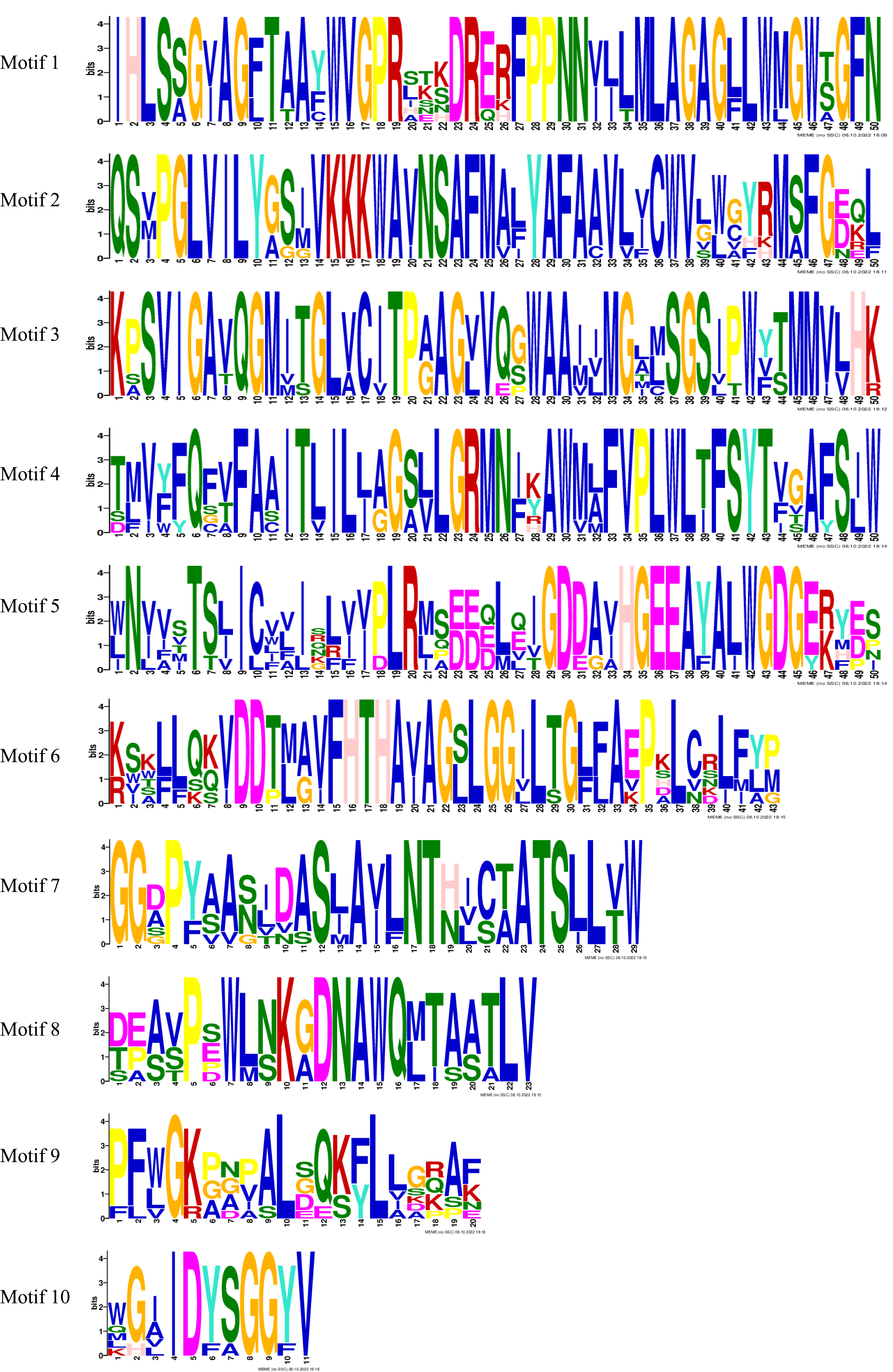

Supplement: Supplementary file 4 [file Image1.JPEG]

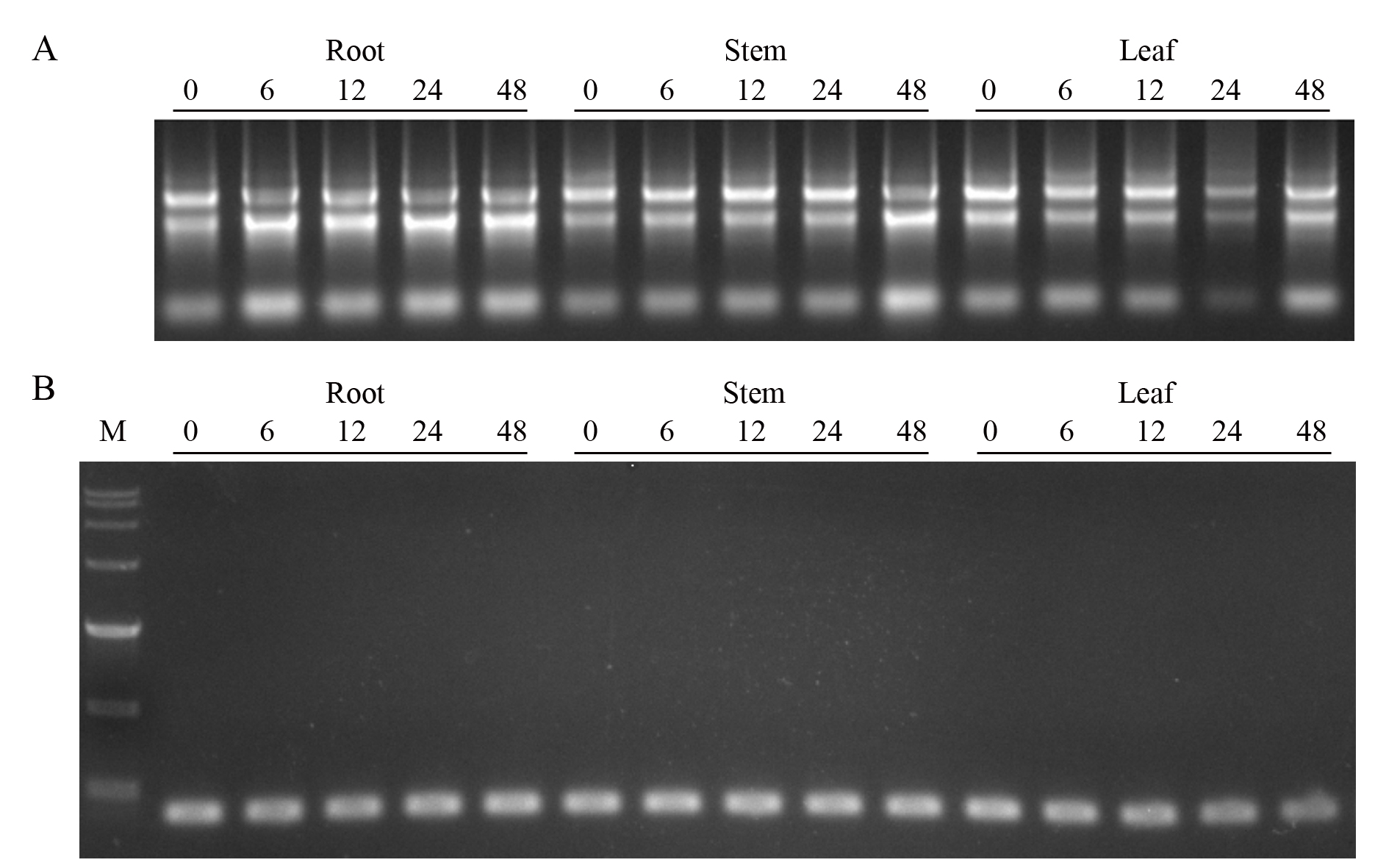

Supplement: Supplementary file 5 [file Image2.JPEG]
