# Supplementary material for: Genome-wide identification, expression profiling, and functional analysis of ammonium transporter 2 (AMT2) gene family in cassava (Manihot esculenta crantz)
Source: Front Genet. 2023 Feb 22;14:1145735. doi: 10.3389/fgene.2023.1145735 (PMC9992417; doi:10.3389/fgene.2023.1145735)
Supplement: Supplementary file 7 [file DataSheet1.docx]

Supplementary Material

**Supplementary Figure S1** The amino acid sequences of each motif identified in MeAMT2 proteins.

**Supplementary Figure S2** RNA extraction (A) and cDNA amplification (B) in cassava roots, stems, and leaves at different time points.

**Supplementary Figure S3** PCR amplification of *MeAMT2.3*, *MeAMT2.5*, and *MeAMT2.6* (A) and enzyme digestion of the recombinant plasmids (B).

**Supplementary Table S1** Primers used in this study.

**Supplementary Table S2** All gene pairs of three different genomes (*Manihot esculenta* vs *Arabidopsis thaliana*, *Manihot esculenta* vs *Glycine max*).

**Supplementary Table S3** Expression of *MeAMT2* genes in different tissues (TPM).
